# Reduction in use of MRI and arthroscopy among patients with degenerative knee disease in independent treatment centers versus general hospitals: a time series analysis

**DOI:** 10.1093/intqhc/mzae004

**Published:** 2024-01-22

**Authors:** Laurien S Kuhrij, Perla J Marang-van de Mheen, Lisanne van Lier, Razia Alimahomed, Rob G H H Nelissen, Leti van Bodegom-Vos

**Affiliations:** Department of Biomedical Data Sciences, Section Medical Decision Making, Leiden University Medical Center, Albinusdreef 2, Leiden 2333 ZA, the Netherlands; Department of Biomedical Data Sciences, Section Medical Decision Making, Leiden University Medical Center, Albinusdreef 2, Leiden 2333 ZA, the Netherlands; Zorg & Zekerheid, Haagse Schouwweg 12, Leiden 2332 KG, the Netherlands; Zorg & Zekerheid, Haagse Schouwweg 12, Leiden 2332 KG, the Netherlands; Department of Orthopaedics, Leiden University Medical Center, Albinusdreef 2, Leiden 2333 ZA, the Netherlands; Department of Biomedical Data Sciences, Section Medical Decision Making, Leiden University Medical Center, Albinusdreef 2, Leiden 2333 ZA, the Netherlands

**Keywords:** quality of health care, low-value care, osteoarthritis, knee

## Abstract

The use of MRI and arthroscopy are considered low-value care in most patients with degenerative knee disease. To reduce these modalities, there have been multiple efforts to increase awareness. Reductions have been shown for general hospitals (GH), but it is unclear whether this may be partly explained by a shift of patients receiving these modalities in independent treatment centers (ITCs). The aims of this study were to assess (i) whether the trend in use of MRI and arthroscopy in patients with degenerative knee disease differs between ITCs and GH, and (ii) whether the Dutch efforts to raise awareness on these recommendations were associated with a change in the trend for both types of providers. All patients insured by a Dutch healthcare insurer aged ≥50 years with a degenerative knee disease who were treated in a GH or ITC between July 2014 and December 2019 were included. Linear regression was used with the quarterly percentage of patients receiving an MRI or knee arthroscopy weighted by center volume, as the primary outcome. Interrupted time-series analysis was used to evaluate the effect of the Dutch efforts to raise awareness. A total of 14 702 patients included were treated in 90 GHs (*n* = 13 303, 90.5%) and 29 ITCs (*n* = 1399, 9.5%). Across the study period, ITCs on an average had a 16% higher MRI use (*P* < .001) and 9% higher arthroscopy use (*P* = .003). MRI use did not change in both provider types, but arthroscopy use significantly decreased and became stronger in ITCs (*P* = .01). The Dutch efforts to increase awareness did not significantly influence either MRI or arthroscopy use in ITCs (*P* = .55 and *P* = .84) and GHs (*P* = .13 and *P* = .70). MRI and arthroscopy uses were higher in ITCs than GHs. MRI use did not change significantly among patients ≥ 50 years with degenerative knee disease in both provider types between 2014 and 2019. MRI- and arthroscopy use decreased with ITCs on average having higher rates for both modalities, but also showing a stronger decrease in arthroscopy use. The Dutch efforts to increase awareness did not accelerate the already declining trend in the Netherlands.

## Introduction

The ‘Choosing Wisely’ (CW) campaign is an initiative to promote conversations between clinicians and patients whether provided care adds value, is evidence-based, and is truly necessary [1]. Overuse of care is not only associated with increased resources and costs but could also potentially harm patients. The CW campaign has spread worldwide, with medical professional societies creating lists of diagnostics and procedures for which there is strong scientific evidence that these do not benefit specific patient groups while having the potential for harm and require significant cost (i.e. low-value care) [1–4].

One of the CW recommendations is not to routinely perform magnetic resonance imaging (MRI) or arthroscopy in patients aged ≥50 years or older with symptomatic degenerative knee complaints [5–7]. Routine MRI use for diagnosis of degenerative knee disease is not recommended for this specific patient group due to the poor association between test results and symptoms. Arthroscopic interventions are not recommended in patients with degenerative knee disease, because there are limited benefits 1–2 years after surgery, except when mechanical symptoms (such as locking symptoms) are present [8]. Therefore, further treatment for most patients with degenerative knee disease should be centered around reducing stress on the joint by weight loss, increasing mobility with physical therapy, and prescribing pain medication if needed [9–11].

So far, adherence to these CW recommendations has been limited [12, 13]. In the Netherlands, about one-third of all arthroscopies performed among patients with degenerative knee disease were considered low-value care [14] with a gradual decrease in MRI and arthroscopy use between 2016 and 2018, likely due to a secular trend [15].

However, only GHs were included in the latter study, leaving out the private sector which we refer to as independent treatment centers (ITCs). Similar to most countries, in the Netherlands, care is outsourced to the private sector to some extent to reduce the stress on general hospitals (GHs). A previous study suggested that private centers deliver more low-value care and less high-value care than GHs [16]. Private centers may continue with low-value care because of financial incentives to perform more diagnostic tests and/or prescribe treatments [17]. Another reason may be that patient expectations are not met in an initial visit to GHs resulting in specific requests for certain tests in a subsequent visit to private centers [9, 18, 19]. In the Netherlands, ITCs are specifically focused to treat low-risk patients with degenerative knee disease who are able to go home on the same day after surgery, whereas GHs also treat higher-risk patients who might require ICU stay or those experiencing complications after surgery in ITCs. In this context, a shift towards these ITCs may have occurred of patients not receiving MRI or arthroscopy in GHs, particularly in case of procedures done at the patient’s request, suggesting that the reduction from a societal perspective may be much smaller than shown for GHs [15].Therefore, this study aimed to assess: (i) whether the trend in MRI and arthroscopy use in patients aged ≥50 years with degenerative knee disease differed between ITCs and GHs, and (ii) whether raising awareness on CW recommendations in the Netherlands was associated with a change in the trend for both provider types.

## Methods

### Study design and setting

This is an observational study using routinely collected reimbursement data from ‘Zorg & Zekerheid’, one of the healthcare insurers in the Netherlands, as only insurance data will include data from ITCs and GHs [20]. ‘Zorg & Zekerheid’ works across the country, but some geographical regions have considerably higher coverage, up to 32% in the northern part of the province South Holland. In the Netherlands, people can choose their own healthcare insurer for (mandatory) basic insurance, add additional insurance if desired, and can even choose their own healthcare providers. Healthcare insurers buy care at various healthcare providers, and in that way, competition is intended to reduce healthcare costs. Almost all care provided by both GHs and ITCs is reimbursed by the insurers, including MRI and arthroscopy and other care for patients with degenerative knee disease. For these patients, a referral by a general physician is necessary to acquire an MRI and/or arthroscopy.

### Data and primary outcome

Patients aged ≥50 years with degenerative knee disease insured at ‘Zorg & Zekerheid’ and a closed care trajectory for Diagnosis Treatment Codes (DTC) 1801 and 1805 between 1 July 2014 and 31 December 2019 treated in a GH or ITC were included. The care trajectory is defined as the first hospital visit for specific symptoms up until the patient is no longer treated or receives follow-up care at that center, with a maximum of 120 days. All consultations, imaging, and treatments related to the DTC are linked to that trajectory. For each patient, we determined whether a MRI was conducted within 90 days after the initial visit and/or an arthroscopy was performed within 6 months, which are the prevailing terms for both entities. Patients with cruciate ligament tears (DTC 1820 and 1830) were not targeted by the CW recommendations and therefore selected as control to assess whether a changing trend in MRI and arthroscopy use was observed.

Center-level data were extracted so that the data could not be traced back to individual patient information (in accordance with the General Data Protection Regulations). The primary outcome was the quarterly percentage of patients receiving MRI and patients receiving arthroscopy, weighted by center volume. Patients were assigned to the quarter in which the care trajectory started. The following data were extracted for each anonymized provider and quarter: DTC, number of patients, number of patients with MRI, number of patients with arthroscopy, type of provider (GH/ITC), and referral status (defined as whether patients receiving MRI/arthroscopy were seen for similar symptoms in a GH before visiting an ITC and vice versa).

### Statistical analysis

All statistical analyses were performed with the R software (version 1.4.1717). A *P* < .05 was used to indicate statistical significance. First, a linear regression model was used to compare GHs and ITCs on the annual number of patients over time and annual crude percentages of MRI and arthroscopy over time.

Then, a linear regression model was used to evaluate the change in quarterly percentage of patients receiving a MRI over time, and similarly for arthroscopy. The quarterly percentage of patients receiving MRI/arthroscopy was weighted by center volume within each provider type, as there is large variation in numbers of patients treated between centers, so that low adherence in a small center may otherwise hardly be taken into account. To evaluate a difference in time trend between ITCs and GHs, the following equation was used: weighted percentage of patients receiving MRI/arthroscopy = α + β1(time) + β2(type of provider)+ β3(time × type of provider). In this equation, ‘time’ was portrayed in quarters and ‘type of center’ indicated whether the patient was treated in a GH or ITC. The interaction term β3 indicates whether the time trend differed between GHs and ITCs.

Subsequently, an Interrupted Time Series (ITS) within each type of provider was performed to evaluate the effect of the efforts made to raise awareness on CW recommendations on MRI/arthroscopy use, in changing the level and/or preintervention time trend. This included a number of initiatives and was primarily directed towards physicians. In November 2016, the report from a nationwide program called ‘To do or not to do?’ by the Dutch Federation of University Medical Centers received a lot of national media attention, stating that MRI and arthroscopy should not be used in patients with degenerative knee disease [21]. On a conference by the Netherlands Orthopaedic Association in February 2017, this statement was further highlighted [6]. For the present study, the first quartile of 2017 was therefore considered as lag time, considering that the intervention would only start to show its effect from the second quartile of 2017 onwards.

For these ITS analyses, the following equation was used: weighted percentage of patients receiving MRI/arthroscopy = α + β1(time) + β2(intervention)+ β3(time × intervention). The intervention compared two periods—before the intervention (Q3 2014 to Q4 2016–10 data points) and after the intervention (Q2 2017 to Q4 2019–11 data points)—with the lag period at Q1 2017 not included in the analysis. The term β1 indicates the time trend before the intervention, and the term β2 the direct effect of the intervention in changing the level of MRI/arthroscopy use. The interaction term β3 indicates the effect of the intervention in changing the preintervention time trend.

No autocorrelation was found in the time series, tested by the Durbin–Watson test, and all trends were stationary which was checked by the Dickey–Fuller test.

We also investigated whether there was a difference in referrals from either GH to ITC or vice versa using a *t*-test for both treatment modalities.

### Sensitivity analysis

To take into account the large variation in number of patients treated between centers, we performed a sensitivity analysis in which all centers with less than 20 patients in a year were excluded for that year, as very small numbers of patients in many centers may influence the results. In addition, we checked for a possible shift in coding towards more patients with another DTC receiving a MRI/arthroscopy after the efforts to raise awareness on CW recommendations. This was done by evaluating changes in coding over time towards patients with similar symptoms but a different etiology (i.e. cruciate ligament tears, DTC 1820 and 1830).

## Results

### Descriptives

In total, 14 702 patients with degenerative knee disease aged ≥50 years were included. Most patients (*n* =13 303, 90.5%) were treated in one of the 90 GHs, of which three patients were referred from an ITC (<1%). The remaining 1399 patients were presented at 29 ITCs, including two patients referred from a GH (<1%). The median number of patients per ITC was 8 (IQR 2–24).

Of all patients, 3066 received an MRI (20.4%) and relatively more patients in ITCs than in GHs (28.4% versus 20.1%), as shown in Table 1. The annual percentage of patients receiving MRI decreased over time, from 24.7% in 2014 to 19.2% in 2019. During the study period, annual MRI use in GHs decreased from 23.1% in 2014 to 18.2% in 2019 and in ITCs from 28.0% to 24.6% in 2019.

**Table 1.** Comparison of patients presented at general hospitals and independent treatment centers and use of MRI and arthroscopy in crude percentages.

	Total	2014 (2nd half)	2015	2016	2017	2018	2019
General hospitals [*n*]	13 303	1108	2326	2412	2447	2535	2475
Independent treatment centers [*n*]	1399	75	156	188	256	298	426
MRI	3066 (20.4%)	277 (24.7%)	569 (22.9%)	565 (21.7%)	581 (21.5%)	518 (18.3%)	556 (19.2%)
General hospitals [*n* (%)]	2669 (20.1%)	256 (23.1%)	518 (22.3%)	500 (20.7%)	509 (20.8%)	435 (17.2%)	451 (18.2%)
Independent treatment centers [*n* (%)]	397 (28.4%)	21 (28%)	51 (32.7%)	65 (34.6%)	72 (28.1%)	83 (27.9%)	105 (24.6%)
Arthroscopy	1221 (8%)	135 (11.4%)	309 (12.4%)	266 (10.2%)	227 (8.4)	153 (5.4%)	131 (4.5%)
General hospitals [*n* (%)]	1082 (8.1%)	114 (10.3%)	280 (12%)	237 (9.8%)	209 (8.5%)	134 (5.3%)	108 (4.4%)
Independent treatment centers [*n* (%)]	139 (9.9%)	21 (28%)	29 (18.6%)	29 (18.6%)	18 (7%)	19 (6.4%)	23 (5.4%)

An arthroscopy was performed in 1221 patients (8%) with relatively more patients receiving an arthroscopy in ITCs than in GHs (9.9% versus 8.1%). The annual percentage of patients receiving an arthroscopy decreased from 11.4% in 2014 to 4.5% to 2019. During the study period, annual arthroscopy use in GHs decreased from 10.3% in 2014 to 4.4% in 2019 and in ITCs from 28% to 5.4% in 2019.

### Time-trend analysis

**Figure 1:**
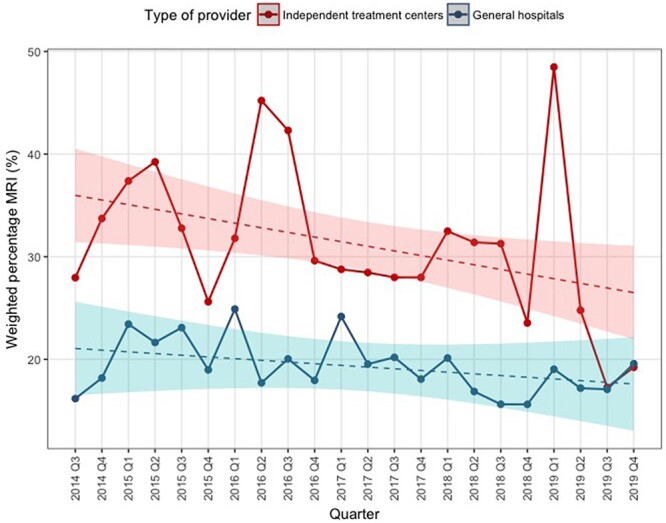
Trend in weighted quarterly percentage of patients receiving MRI for independent treatment centers versus general hospitals.

Figure 1 shows the trends in weighted quarterly percentage of patients receiving MRI in ITCs versus GHs during the study period. ITCs on average had 16% higher MRI use than GHs (*P* < .001, Table 2). The decreasing trend in MRI use was neither statistically significant (*P* = .29) nor different between the provider type (*P* = .22). The efforts to raise awareness on the CW recommendations (intervention) did not result in a significant change in level of MRI use for either ITCs (*P* = .55) or GHs (*P* = .84) nor did it change the trend over time (*P* = .30 and *P* = .58, respectively), as shown in Table 3.

**Table 2.** Results of the time series analysis for the weighted quarterly percentage of patients receiving either an MRI or arthroscopy, comparing the trend over time in independent treatment centers to public hospitals.

	MRI	Arthroscopy
Parameter	Estimate (SE), *P*-value	
Intercept	0.22 (0.02), *<.001*	0.14 (0.02), *<.001*
Time (in quarters)	−0.002 (0.002), *.29*	−0.005 (0.002), .002^*^
Type of provider (ITC^ vs general hospital)	0.16 (0.03), <.001^*^	0.09 (0.03), .003^*^
Time*Type of provider	−0.003 (0.002), *.22*	−0.006 (0.002), .01^*^

*P-value statistically significant < 0.05. ^ITC = independent treatment centers.

**Table 3.** Results of the time series analysis for the weighted quarterly percentage of patients receiving either an MRI or an arthroscopy, comparing the effect of the intervention within independent treatment centers (ITC) and general hospitals. Pre-intervention period is Q3 2014 – Q4 2016, post-intervention period is Q2 2017 – Q4 2019 with Q1 2017 as lag time excluded from the analyses.

	MRI		Arthroscopy	
	ITC	General hospital	ITC	General hospital
Parameter	Estimate (SE), *P*-value			
Intercept	0.32 (0.05), *<.001*	0.20 (0.02), *<.001*	0.27(0.04), *<.001*	0.15 (0.008), *<.001*
Time (in quarters)	0.005 (0.008), *.55*	0.0004 (0.003), *.89*	−0.02 (0.007), .03^*^	−0.005 (0.001), .001^*^
Intervention (post versus pre)	0.08 (0.13), *.55*	0.009 (0.04), *.84*	−0.18 (0.11), *.13*	−0.009 (0.02), *.70*
Time*Intervention	−0.01 (0.01), *.30*	−0.002 (0.004), *.58*	0.01(0.009), *.15*	0.001 (0.002), *.70*

*P-value statistically significant < 0.05.

**Figure 2:**
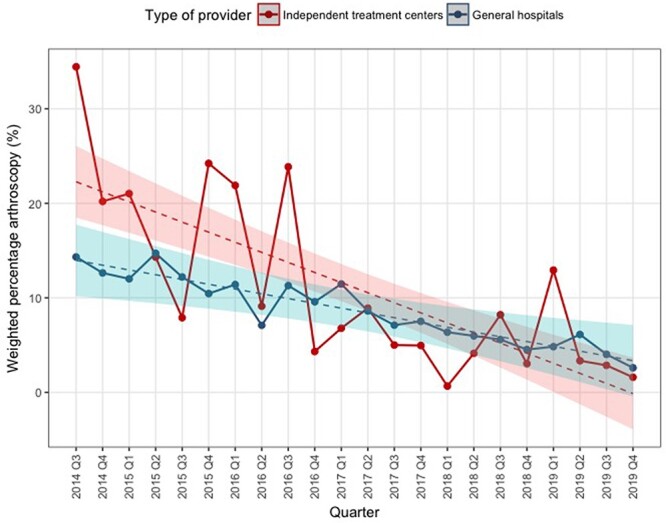
Trends in weighted quarterly percentage of patients receiving arthroscopy for independent treatment centers versus general hospitals. Q = quarter.

Figure 2 shows the trends in weighted quarterly percentage of patients receiving an arthroscopy, in ITCs versus GHs. ITCs on average had 9% higher arthroscopy use than GHs (*P* = .003, Table 2). Arthroscopy use significantly decreased by 0.5% per quarter (*P* = .002) and differed between provider type with ITCs having a stronger reduction than GHs (*P* = .01). The efforts to raise awareness on the CW recommendations did not change the level of arthroscopy use (ITCs *P* = .13, GHs *P* = .70) nor the trend (ITCs *P* = .15, GHs *P* = .70), as shown in Table 3. Also shown is the significantly decreasing preintervention trend in both ITCs (*P* = .03) and GHs (*P* = .001).

### Sensitivity analysis

The sensitivity analysis including only centers with at least 20 patients in a year shows a similar higher MRI use in ITCs than in GHs (*P* < .001, Supplementary Table 1). For arthroscopy use, the same significantly declining trend over time was found, but ITCs no longer had higher arthroscopy use than GHs (*P* = .08) nor was the decrease over time stronger in ITCs (*P* = .11, Supplementary Table 2).

Another sensitivity analysis was performed to check for a possible shift in coding associated with the intervention. Out of the 137 patients with cruciate ligament tears, postintervention MRI use decreased from 27 out of 45 patients to 28 out of 59 patients in GHs and from 4 out of 5 patients to 19 out of 28 patients in ITCs. Arthroscopy use decreased from 2 out of 45 to 3 out of 59 in GHs, with no arthroscopy performed pre- or postintervention in ITCs. Given the small numbers, it is unlikely that a shift in coding practices towards using the DTC of cruciate ligament tears explains the observed declining trend in MRI/arthroscopy use for patients with degenerative knee disease.

## Discussion

### Statement of principle findings

This study shows that the percentage of patients with degenerative knee disease receiving MRI did not change between 2014 and 2019 in both GHs and ITCs, while the percentage of patients receiving an arthroscopy was significantly reduced and more so in ITCs. On average, the percentage of patients receiving MRI/arthroscopy was significantly higher in ITCs than in GHs, which might explain part of the stronger reduction in arthroscopy use over time in ITCs to arrive at similar levels as GHs. The Dutch efforts to raise awareness on CW recommendations did not influence the level and/or trend in either GHs or ITCs; for arthroscopy the preintervention trends were already declining significantly.

### Strengths and limitations

The strength of our study is that we used data from a healthcare insurer so that our results are not based on the policy of one healthcare provider or region but reflects all patients insured through the insurer at GHs and ITCs across the country. Additionally, MRI and arthroscopy for this patient group are reimbursed by all healthcare insurers, and this is likely a representative sample of patients. Also, we assessed that code-shifting, i.e. changing codes to an unjust diagnosis with similar symptoms as shown in a previous study by Hamilton *et al*. [21], could not explain our findings.

A limitation of our study is that we have no data on specific symptoms, such as patients with locking symptoms or with knee complaints due to trauma, for whom, respectively, arthroscopy and MRI are warranted. However, it seems unlikely that the percentage of these patients would be distributed differently between GHs and ITCs as it was similar in a previous study by Rietbergen et al [15]. So, it seems unlikely to affect our conclusions on differences between GHs and ITCs.

### Interpretation within the context of the wider literature

A nationwide survey in Norway previously showed higher use of MRI services for knee pain in private facilities versus public hospitals in 2002–2004 [22]. Unlike the Netherlands, healthcare provided by private clinics in Norway is only publicly reimbursed when the provider is contracted by the national healthcare system. For arthroscopy use, previous studies in France and Canada, both with similar reimbursement policies for the private sector as the Netherlands, have also shown significantly higher arthroscopy use in private centers with arthroscopy use decreasing over time [23, 24]. However, the previous studies were all conducted before the CW campaigns to reduce this low-value care and could therefore not capture the additional effect of these campaigns to pre-existing time trends. The current study therefore adds that even after the CW and other similar campaigns, particularly MRI use is still higher in ITCs than GHs for this patient group.

The present study found no statistically significant effect associated with the efforts to raise awareness for CW for both ITCs and GHs, consistent with previous studies showing limited effect of similar interventions in GHs [7, 8]. It is important to note that the preintervention trend in arthroscopy use already declined significantly, meaning that the intervention did not have an additional effect on top of this secular trend. The lack of an effect may be interpreted as that merely raising awareness or educational interventions may not be sufficient to reduce low-value care [25]. Another issue is that clinician factors (such as the fear of missing a diagnosis), patient preferences, and organizational factors (such as culture and reimbursement policies) make de-implementation of low-value care difficult [26, 27]. For instance, a rationale to still perform low-value care is the doctors’ belief that patients will go to an ITC if they do not receive a test. However, in our study, we hardly found any referrals and therefore no indication to support this belief.

### Implications for policy, practice, and research

Our study shows that solely efforts to raise awareness to reduce low-value care are not sufficient. In terms of what might be effective alternative interventions to reduce low-value care, two systematic reviews showed that a multicomponent intervention using clinical decision support and performance feedback along with a change in payment policy might be promising [28, 29]. Another intervention worth considering is the use of performance indicators to monitor low-value care use as a part of continuous feedback, which is currently not available in the Netherlands for MRI/arthroscopy use among the patient group studied here. Through the nationwide program ‘Health Care Evaluation and Appropriate Use’, such indicators are being introduced to reduce low-value care [30]. Future research should reveal which of these interventions are most effective in reducing low-value MRIs and arthroscopies. A multicomponent intervention to reduce this low-value care is necessary to increase the quality of care delivered to these patients.

### Conclusions

MRI and arthroscopy use in patients with degenerative knee disease have decreased over the years, but MRI use remains significantly higher in ITCs compared with GHs. Although arthroscopy use was higher at the start of the study period, the observed stronger reduction over time resulted in reaching similar use in both provider types at the end of the study period. More surveillance not only for GHs but also for ITCs is necessary to detect low-value care. Efforts to raise awareness have been launched to inform healthcare providers as well as patients to reduce this care, but these interventions did not have a significant effect to further decrease the already pre-existing declining trend.

## Supplementary Material

mzae004_Supp

## Data Availability

Data are not publicly available due to privacy regulations of Zorg & Zekerheid and agreements as specified in the contract. A request for access to the study data can be submitted to Zorg & Zekerheid.
