# Supplementary material for: Reduction in use of MRI and arthroscopy among patients with degenerative knee disease in independent treatment centers versus general hospitals: a time series analysis
Source: Int J Qual Health Care. 2024 Jan 22;36(1):mzae004. doi: 10.1093/intqhc/mzae004 (PMC10849166; doi:10.1093/intqhc/mzae004)
Supplement: mzae004_Supp [file mzae004_supp.zip › suppl_data/Supplement 2.docx]

| **Parameter** | **Estimate (SE)** | **P-value** |
| --- | --- | --- |
| Intercept | 0.14 (0.02) | < 0.001 |
| Time (in quarters) | -0.005 (0.001) | 0.002 |
| Type of provider (ITC^ vs general hospital) | 0.06 (0.03) | 0.08 |
| Time*Type of provider | -0.04 (0.002) | 0.11 |

**Supplemental table 2.** Results of the sensitivity analysis for the weighted quarterly percentage of patients receiving an arthroscopy. In this sensitivity analysis, centers treating less than 20 patients in a year were excluded for that year. ^ITC = independent treatment center.
